# Perspectives on Early Power Mobility Training, Motivation, and Social Participation in Young Children with Motor Disabilities

**DOI:** 10.3389/fpsyg.2017.02330

**Published:** 2018-01-09

**Authors:** Hsiang-Han Huang

**Affiliations:** ^1^Department of Occupational Therapy, Graduate Institute of Behavioral Sciences, Chang Gung University, Taoyuan, Taiwan; ^2^Department of Physical Medicine and Rehabilitation, Chang Gung Memorial Hospital, Linkou, Taiwan

**Keywords:** independent mobility, motivation, social participation, young children with motor disabilities, power mobility devices

## Abstract

The efficacy of traditional training programs (e.g., neurodevelopmental therapy) in promoting independent mobility and early child development across all three International Classification of Functioning, Disability, and Health levels lacks rigorous research support. Therefore, *early* power mobility training needs to be considered as a feasible intervention for very young children who are unlikely to achieve independent mobility. This perspective article has three aims: (1) to provide empirical evidence of differences in early independent mobility, motivation, daily life activities, and social participation between young children with typical development and motor disabilities; (2) to discuss the contemporary concepts of and approaches to early power mobility training for young children with motor disabilities and the current need for changes to such training; and (3) to provide recommendations for early power mobility training in pediatric rehabilitation. Independent mobility is critical for social participation; therefore, power mobility can be accessible and implemented as early as possible, specifically for infants who are at risk for mobility or developmental delay. To maximize the positive effects of independent mobility on children’s social participation, early power mobility training must consider their levels of functioning, the amount of exploration and contextual factors, including individual and environmental factors.

## Introduction

Researchers have recently focused on reducing the limitations of young children with motor disabilities in social, cognitive, perceptual, and functional development induced by their early lack of independent mobility (Butler, 1986; Lynch et al., 2009; Ragonesi et al., 2010; Livingstone and Field, 2014; Livingstone and Paleg, 2014). However, in the last 20 years, the lack of rigorous studies and limited evidence of the efficacy of traditional walking training programs (e.g., neurodevelopmental therapy-NDT) in promoting independent mobility and early child development across all three International Classification of Functioning, Disability and Health (ICF) (World Health Organization, 2001) levels indicate the need to consider an effective way to promote efficient independent mobility and early development across all three ICF levels, particularly the psychosocial aspects (Bray et al., 2014).

Independent locomotion plays a vital role in psychological development (Anderson et al., 2013). Recent studies have investigated functioning in other areas including social capabilities and participation in children with cerebral palsy (CP) due to the non-motor neurodevelopmental issues that often accompany CP, such as sensation, perception, and communication disturbances (Rosenbaum et al., 2007; Whittingham et al., 2010; Konst et al., 2014). There is increasing evidence for the positive impact of power mobility devices (PMDs) use on such children’s overall development (Livingstone and Field, 2014, 2015). To ensure acquisition of exploratory experience as closely as children with typical development (TD), proponents of PMDs for very young children have presented several compelling reasons for introducing power mobility training to children when children with TD engage in independent mobility (Livingstone, 2010; Jones et al., 2012; Larin et al., 2012; Livingstone and Field, 2014). With the developing research-based evidence in the recent years, early power mobility training is suggested as a feasible intervention in clinical settings for very young children who have not achieved independent mobility and are unlikely to do so (Wiart and Darrah, 2002; Livingstone and Field, 2014; Livingstone and Paleg, 2014; Morgan et al., 2016).

Livingstone and Field (2014) systematic review and the Delphi report (2014) have provided the body of evidence and practice considerations for early power mobility training and possible effects on overall development. However, most evidence of early power mobility training (i.e., participants were younger than 3-years-old) in the systematic review focused on initiation of mobility, driving skills development, and the impact on functional mobility skills (Livingstone and Field, 2014). Few studies included outcomes related to motivation and social skills and tended to be observational and descriptive. Livingstone and Paleg (2014) have suggested that power mobility may address the secondary effects on other areas of development as socialization for very young children who do not move and explore independently; nevertheless, evidence regarding social skills was limited and weak.

Therefore, this perspective article highlights the importance of early independent mobility, motivation, exploratory learning, and social participation based on ecological theory and the ICF model, and addresses the major differences between young children with TD and children with motor disabilities. In addition, more evidence related to the use of PMDs and changes on motivation and socialization in young children with motor disabilities is included. The application of early power mobility training using a family-centered, context-focused approach is further proposed for enhancing independent mobility and overall development of children with motor disabilities, particularly social development and participation. This study has three specific aims: (1) presenting the contemporary theory on early development and outlining empirical evidence on the differences in early independent mobility, daily life activities, and social participation between young children with TD and children with motor disabilities; (2) discussing the contemporary concepts of/approaches to early power mobility training for children with motor disabilities and the need for changes in such training; and (3) providing recommendations from contemporary perspectives for implementing early power mobility training to improve independent mobility and socialization in pediatric rehabilitation.

## Early Independent Mobility and Specific Environments as Facilitators of Development/Social Participation

### Contemporary Perspective on Early Development

**FIGURE 1:**
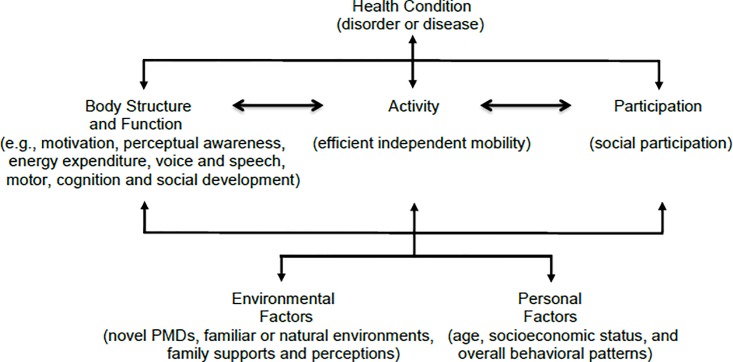
The World Health Organization international classification of functioning, disability, and health (2001).

The conventional perspective on early development adopted by many clinicians is heavily influenced by the neuromaturational theories of Gesell (1946) and McGraw (1946), which stressed the importance of neuromuscular maturation relative to practice and experience in explaining developmental change. Maturation and the role of learning are impossible to parse apart during the developmental process (Thelen, 2000). Children benefit from experience when they have the neurological and muscular maturity (Gottlieb, 1998). The contemporary perspective proposed by Gibson’s ecological theory (2000) views development as multicausal, including perceptual information, infants’/children’s capabilities, their biomechanical changes, and their environments’ physical properties. The core concept closely relates to dynamic systems theory development in developmental science (Thelen and Smith, 1994; Spencer et al., 2011) and is consistent with the ICF model (World Health Organization, 2001) that emphasizes the dynamic reciprocal relations between the functioning levels (body function/structure, activity, and participation) and contextual factors (personal, environmental) (**Figure 1**).

**FIGURE 2:**
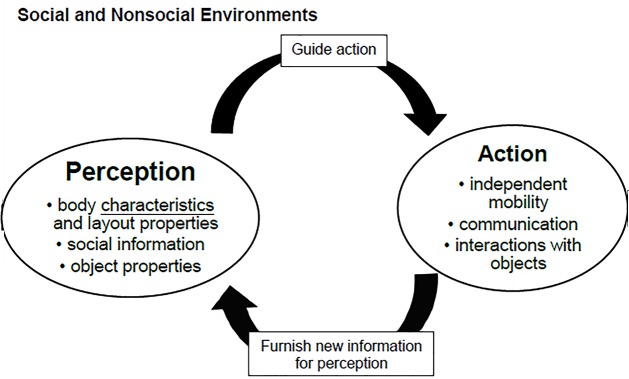
Perception-action coupling and environment. On the basis of Gibson’s ecological approach to perceptual learning and development (Gibson and Pick, 2000).

Ecological theory has focused on how infants and children learn to perceive affordances through perception-action coupling within the environment during early development (Bertenthal, 1996; Gibson and Pick, 2000). Affordance is the fit between an actor and environmental supports, which enable performing an action (Gibson, 1979). To perceive affordances, information from the environment or child must be scaled relative to each other and explored through perception-action cycles within the environment (Warren, 1984; Huang et al., 2013). Perception and action changes are cyclic and repetitive, enabling infants/children to improve the fits between their own action capabilities, their bodies’ perception, and the environment layout continually (**Figure 2**).

As an intrinsic body function, motivation enables an individual to autonomously and consistently perform and enjoy activities with moderate difficulty (Morgan et al., 1995; Turner and Johnson, 2003). Motivation for making environmental contact and obtaining information (exploratory activity) spontaneously provides the foundation for learning affordances during the early development process (Gibson and Pick, 2000). This learning requires exploration as practice that is not merely repeating one specific solution, but various solutions to one goal. Active exploration helps gather different experiences and information about object-surface properties to find the optimal way of gaining behavior control within the environment (Newell, 1996; Adolph and Kretch, 2015). Locomotion, one of the three major developing behavior systems during the first 3 years, facilitates affordances learning, such as the affordances of other’s gestures, facial expressions, vocalizations, object properties, learning to control the whole body to move around, and extending the ability for using affordances and resources (Gibson and Pick, 2000).

Both ecological theory and the ICF model illustrate how the person-environment interaction in infants’ and young children’s daily lives shapes and defines their health and well-being. For promoting early development health, child-environment fit involving multiple persons and environmental factors and developing the three behavior systems must be focused. Locomotion as independent mobility is one action system linked to the environment affecting other developmental domains, particularly the social one (Gibson and Pick, 2000).

### Independent Mobility for Children with TD Younger Than 3 Years Old

The onset of independent mobility enables young children to explore more and learn to acquire specific information from their non-social and social worlds (Gibson and Pick, 2000). In the non-social world, independent mobility allows understanding their environment layout (e.g., becoming aware of heights, avoiding obstacles, and determining an object’s unique properties) (Gibson, 1988; Anderson et al., 2013; Huang et al., 2013). Adolph (2005) and Adolph et al. (2012) conducted a series of studies to obtain details of infants’ everyday experiences with balance control during stance and locomotion. They found that infants with TD took approximately 2368 steps (the length of 7.7 American football fields) and had 17 falls/h. Every step provided practice by coordinating segments of the leg, stabilizing the trunk and head, gathering and using perceptual information of environment, and generating anticipatory and compensatory responses to balance loss (Adolph et al., 2012). Independent mobility widens their exploration and enhances their motor, perceptual, and cognitive development (Gibson and Pick, 2000).

In the social world, independent mobility provides children more opportunities for parental- or peer-interaction and engagement in quality play (Smith et al., 2011; Guerette et al., 2013). Such experiences help children to begin exploring social information such as facial expressions, gestures, and vocalizations, which promote self-motivated behavior (Gibson and Pick, 2000). Beyond furthering the social experience attainment, early independent mobility promotes infants’ sense of security, enriches their emotional life, and helps them learn behaving before others (Gustafson, 1984; Gibson and Pick, 2000; Clearfield, 2011). The onset of independent mobility is an indispensable contributor to early childhood social development (Campos et al., 2000).

There are few studies examining young children’s time usage and daily activities—useful for determining the amount of independent mobility required for a typical child every day (Hofferth and Sandberg, 2001; Ben-Arieh and Ofir, 2002). On weekdays, children with TD younger than 3 years spend an average of 3.8 h/day performing physical activity (Hofferth and Sandberg, 2001; Hnatiuk et al., 2012), and might spend another 3.8 h/day on social roles involving social participation (e.g., attending daycare, visiting friends, or engaging in personal care and hobbies with others) (Hofferth and Sandberg, 2001). Generally, young children spend at least one-third of their day playing, moving freely in various environments, and in social participation (Hofferth and Sandberg, 2001; Ben-Arieh and Ofir, 2002). This considerable exploration allows them to perceive what family members, peers, and the neighborhood layout offer that relates to their independent mobility, motivation, and communicative ability, (i.e., the child-environment fits) (Kenney, 2012). Early development is connected to exploration, the health condition proposed by the ICF model involving functioning levels (motivation, independent mobility, and social participation), and contextual factors (accessible environments and family characteristics).

### Independent Mobility for Young Children with Motor Disabilities

Young children with motor disabilities (e.g., CP, developmental delay) and restricted mobility due to neuro-muscular disorders often display more passive behaviors with decreased curiosity, initiative, and motivation (Tatlow, 1980). Immobility limits a child’s exploratory experience, leading to a condition called “learned helplessness,” wherein children give up trying to control their own world and become dependent on others. They are incapable of learning to perceive the affordances of other’s actions and intentions directed toward oneself and responding to others adaptively. This condition, according to Godfrey et al. (2002), can last well into adulthood, with teenagers with disabilities being twice as likely as healthy teenagers not being involved in education, employment, or training.

Compared to young children with TD, there is very limited research on daily time spent on play and social activities for young children with motor disabilities. What little we do know is that lower levels of gross motor functioning abilities are associated with poorer social ability in young children with CP (Whittingham et al., 2010; Konst et al., 2014). Whittingham et al. (2010) suggested “independent functional mobility may play a key role in social development” (p. 1350). The continuous failure to explore and master everyday situations may relate to developing a self-perception of incompetence and passive resignation that extinguishes further attempts (Burhans and Dweck, 1995). Indeed, the most frequent outcome of restricted self-initiated locomotion is a pattern of passive, dependent behavior—specifically, a lack of curiosity and initiative that persists into later life (Livingstone and Field, 2014). Taking together the ecological theory and the ICF model (Gibson and Pick, 2000; World Health Organization, 2001), these previous findings suggest that early intervention programs should focus on promoting a healthy condition affected by independent mobility, motivation, and social participation in young children with motor disabilities (**Figure 1**).

## Current Approaches to Increase Independent Mobility in Early Intervention

### Changing View of Early Mobility Training and the Use of PMDs

Evidence suggests that independent mobility may improve numerous perceptual and social skills in children with motor disabilities, including spatial awareness and visual perception, spontaneous vocalizations, initiation of contact with others, motivation to explore, and ability to interact meaningfully with peers (Livingstone and Field, 2014, 2015; Livingstone and Paleg, 2014; Sonday and Gretschel, 2016). Based on neuromaturational theory, the conventional view of early independent mobility training focuses on applying neurological approaches (e.g., NDT) and emphasizes the acquisition, use, and maintenance of normal walking patterns as the primary goals, rather than “function” and “efficiency” of a child’s daily life activities focused on increasing exploration within various environments (Shumway-Cook and Woollacott, 1995). However, this conventional perspective has been challenged recently due to convincing evidence suggesting that NDT does not improve contracture and tone in children with motor disabilities (Novak et al., 2013). Furthermore, the evidence for NDT influencing functional motor gains on children with motor disabilities is weak.

Contemporary theories like ecological theory and dynamic systems theory have emphasized functionality, contextual factors, active exploration, and participation rather than normalizing motor patterns in early development (Perry, 1998; Anaby et al., 2016). The contemporary perspective focuses more on activity and participation levels and considers contextual factors as mediators and moderators of treatment effects (Darrah et al., 2011; Lobo et al., 2013). It posits that children’s practicing normal walking patterns is not always the only movement solution—providing diverse movement options for independent mobility for optimal exploration is necessary. Using PMDs, which aim to provide efficient independent mobility and alter the contextual factors, may help increase exploration and improve the three ICF functioning levels of young children with motor disabilities (Henderson et al., 2008; Livingstone and Field, 2014).

Several studies have demonstrated that early power mobility training is feasible for children aged younger than 3 years, even those who might be categorized as “not-ready” based on motor or cognitive abilities (Livingstone and Field, 2014, 2015; Logan et al., 2016). Livingstone and Paleg’s (2014) Delphi study provided a practical consideration of power mobility training for young children with disabilities, and suggested acquisition of mobility experiences as early as 8 months of age. None of these studies reported any safety or physical development problems due to PMD use (Livingstone and Field, 2014). Furthermore, they provided evidence for improved vocalizations, arm and hand movements, motivation to explore, self-confidence and curiosity, and the positive effects on children’s families (Livingstone and Field, 2014, 2015). PMDs may promote the emergence of efficient independent mobility yielding perceptual information of non-social and social environments, resulting in a continuous perception-action cycle.

### The Application of Early Power Mobility Training in Daily Life and Its Impacts on Motivation and Socialization

With the increased independent mobility using PMDs, young children with motor disabilities can engage with their various environments more than before, and begin noticing social norms (Bottos et al., 2003; Ragonesi et al., 2010). Ragonesi and Galloway (2012) suggested that the experience of social interaction obtained by using PMDs might help young children with disabilities perceive people’s responses to their actions and attend to their own actions, thereby changing their social behaviors. Some studies have provided evidence of improvements in play and social skills for young children with motor disabilities after using the PMDs (Livingstone and Field, 2014; Logan et al., 2016; Huang et al., 2017). However, most research used behavioral observations or unstandardized measurements of parents’ survey to assess children’s social interactions (Deitz et al., 2002; Home et al., 2003; Ragonesi et al., 2010, 2011; Ragonesi and Galloway, 2012). Moreover, no studies examined the effects of PMDs use on motivation. There is increasing evidence of early power mobility training in recent years, which focuses on the outcomes of motivation and socialization by using standardized measurements, including the Dimensions of Mastery Questionnaire, the pediatric evaluation of disability inventory and its’ computer adaptive test (Logan et al., 2014; Huang and Chen, 2017; Kenyon et al., 2017a,b). These studies showed the use of laboratory-designed PMDs or modified ride-on cars (ROCs) might increase the child’s motivation to master interpersonal tasks and result in positive changes on social functioning.

Livingstone and Field (2014) have emphasized the need for development of PMDs to enhance environmental interaction. The application of alternative PMDs or modified ROCs may be beneficial for addressing the concerns expressed by parents regarding the cost, size, weight, transportation, and adjustability (Huang and Galloway, 2012). Furthermore, due to these features, the devices may provide various physical environments that may promote children’s motivation to move, play, and interact with people (Huang et al., 2014, 2017; Waldman-Levi and Erez, 2015; Logan et al., 2016). However, Huang and Chen (2017) found that although social function had increased within a treatment group engaged in the ROC training, there were no significant differences between this group and the regular therapy group. Studies indicated that social play, maternal didactic interaction, and caregivers’ perceptions of children’s motivation may relate to preschoolers’ object and social mastery motivation (Jennings et al., 1988; Hauser-Cram, 1996; Wang et al., 2013, 2014; Medeiros et al., 2016). To enhance motivation and social participation, using PMDs in early mobility training may require exploratory activities related to social interaction and children’s developmental levels, creating a social learning environment. Adding social elements, increasing parenting beliefs and parent–child interaction, and adapting the physical and social environment during power mobility training may be an effective way to induce or maintain children’s mastery motivation and increase social interaction (Waldman-Levi and Erez, 2015; Medeiros et al., 2016). The application of PMDs for very young children may be incorporated into a program that considers the exploratory activities relevant to socialization and motivation, individual contextual factors (e.g., developmental level), and environment (e.g., emotional support, adequate space to explore).

### Early Power Mobility Training and a Family-Centered, Context-Focused Approach to Enhance Exploration and Social Participation

Combining early power mobility training with a family-centered, context-focused approach is an emerging contemporary perspective of rehabilitation for young children with disabilities (Darrah et al., 2011). The family-centered services model, emphasizing coaching and cooperation with caregivers, might increase the treatment effects and help children apply their learned skills to their natural environments (Bailey et al., 1986). Interventions based on this approach focus on improving children’s motor-based functional activities primarily by promoting exploration with family support and changing task or environment factors (Darrah et al., 2011; Law et al., 2011). The family is involved in intervention planning by providing unique information about the child and their preferences for the therapy direction (family perceptions). The therapist aims to develop intervention plans that motivate the child and satisfy the family. Both the child and family have active roles in finding solutions for motor and daily activity problems (family support).

Three general principles related to the application of this family-centered, context-focused approach (Darrah et al., 2011) can be integrated into an early power mobility training program to enhance independent mobility and social participation: encouraging exploratory social learning, promoting self-initiated movements, and providing various social and physical environments. First, the caregivers/therapists may allow sufficient time for the child to process, explore, react, and engage in problem-solving (Huang and Galloway, 2012). The delayed response to provide assistance and verbal reinforcement may be applied as social environment to promote children’s mastery motivation during the intervention (Waldman-Levi and Erez, 2015). The caregivers’ and therapists’ roles include encouraging exploration and organizing the environment to promote independent mobility and socialization, rather than providing directive, predominant guidance. During the initial phase of learning to move, creating a social environment for exploration (i.e., one-on-one instruction with distinct gestures, facial expressions, vocalizations, and responsiveness) can be applied to directly guide the child to perform desired social behaviors (e.g., greeting, smiling, verbalizations, or gestures for help). To enhance active, exploratory-oriented learning, adults may include various encouragements (e.g., clapping, exaggerated positive facial expressions) to compliment the child’s achievements, as a positive reinforcement.

Second, to gain independent mobility control through PMD use and repeated percept-action cycles, training may emphasize the power mobility learning’s early phases by addressing the personal factors and child’s functioning levels. Rather than immediately identifying the child’s prerequisite skills, early power mobility training may begin with the child learning the resources and consequences of self-produced movements. The only prerequisite needed for such training might be the desire for mobility and acting on the environment, that is, motivation. Mastery motivation, as a function of the body, cannot only influence children’s behaviors and performances in both family and educational spheres, but can also increase or decrease as mediated by environmental and personal factors (Sparks et al., 2012; Miller et al., 2016). Personal factors (e.g., child’s developmental age and behavioral patterns related to the postural stability level) affect the child’s willingness to learn power mobility skills and persist during challenging tasks (Wang et al., 2011; Livingstone and Paleg, 2014; Miller et al., 2015). Children’s initial PMD use should correspond with age-appropriate and developmental expectations, and use adequate postural support to enhance their ability to use their hands actively to explore. With the reciprocal relationship between children’s functioning and motivation, children with greater persistence are more engaged and directed to produce self-initiated movements for obtaining environmental information in relation to their own actions.

Third, training should be provided in different physical and social environments to ensure that children learn to perceive relevant information in the social and non-social worlds based on the ecological theory (Adolph and Kretch, 2015). Sequentially presenting the environmental layout is beneficial for children to learn to perceive these fits during the training phase. Initial training might occur in a set-up environment within a familiar location with interest areas and verbal reinforcement (Waldman-Levi and Erez, 2015), for example, child’s home with favorite toys, which may induce their motivation and help them gather relevant information about their own movements; PMD’s properties; and other’s reactions, such as understanding the causal effects of self-produced movements, relationship between the PMD’s control system (e.g., a switch) and the device’s motion, and different methods of expressing emotion and responding (Jones et al., 2012; Huang et al., 2014; Logan et al., 2014). The familiar environment allows children to explore themselves and PMD functions spontaneously, which is beneficial to foster their autonomy and influence motivation (Waldman-Levi and Erez, 2015). After learning to control the PMDs for independent mobility, different physical and social environments (e.g., park, playground) may be introduced to enhance the child’s exploratory behaviors and learn a variety of information through self-produced mobility and their environments. Some peer interaction or social games can also be integrated into the functional activities, thus allowing engagement in social play (e.g., sharing toys or tools with other children while driving at the playground). This element of interpersonal contact can increase the child’s sustainability and pleasure, i.e., motivation to perform these activities.

## Conclusion

Given independent mobility’s critical nature for motivation and social participation, power mobility training can be accessible and implemented as early as possible, particularly for infants at risk of mobility or developmental delay. PMDs can be used as compensatory tools to achieve activity or participation goals and training tools for developing body functions and structures. Early power mobility training should consider exploratory learning, children’s functioning, and contextual factors together, which refers to applying a family-centered, context-focused approach. Future research may focus on examining the roles of exploration and contextual factors in early power mobility training, including the amount of practice, different developmental levels, various physical or social environments, family perceptions and the amount of family support, to understand how they moderate and mediate treatment effectiveness on motivation and socialization. The long-term effects of early power mobility training with novel PMDs, such as modified ROCs, on motivation and social participation, should also be examined because of enhanced perception-action cycling. Further rigorous studies should determine the effects of the novel use of early power mobility training across all ICF levels as a holistic therapeutic intervention for children with mobility impairments.

## Author Contributions

H-HH was responsible for organizing, articulating, writing and submitting this manuscript.

## Conflict of Interest Statement

The author declares that the research was conducted in the absence of any commercial or financial relationships that could be construed as a potential conflict of interest.
